# Group model-building approach to understand the dynamics and identifying entry points to build resilience in the groundnut value chain in Senegal

**DOI:** 10.3389/fnut.2026.1741094

**Published:** 2026-06-25

**Authors:** Shalander Kumar, Abhishek Das, Adjani Nourou-Dine Yessofou, Omonlola Nadine Worou, Fama Gueye, Assane Beye, Akinseye Folorunso, Anthony Whitbread

**Affiliations:** 1 International Crops Research Institute for the Semi-Arid Tropics, Hyderabad, India; 2 International Crops Research Institute for the Semi-Arid Tropics, Dakar, Senegal; 3 International Livestock Research Institute, Dakar, Senegal; 4 Faculty of Economics and Management, Cheikh Anta Diop University, Dakar, Senegal; 5 International Livestock Research Institute, Dar es Salaam, Tanzania

**Keywords:** groundnut value chain, Group Model Building, policy, resilience, Senegal, food security, West Africa

## Abstract

**Introduction:**

Senegal’s groundnut value chain covers approximately 1.25 million hectares and underpins the food security and livelihoods of a large rural population. Yet it is beset by persistent structural constraints across both supply and demand dimensions, including weak seed systems, fragmented input delivery, volatile prices, low processing capacity utilization, and climate-driven production risks, that limit its efficiency, inclusiveness, and resilience.

**Methods:**

This study combined large-scale primary data collection with a participatory Group Model Building (GMB) approach. A baseline household survey was conducted with 503 groundnut farmers across 18 villages in the peanut basin of Senegal (regions of Kaffrine, Louga, and Thiès), supplemented by 20 Focus Group Discussions and Key Informant Interviews with processors, traders, government representatives, cooperatives, and research institutions. Two GMB workshops were facilitated in Dakar in March and April 2023, convening 14 participants representing diverse value chain stakeholders. Causal Loop Diagrams were developed iteratively through structured elicitation, plenary validation, and post-workshop verification to map feedback structures and identify leverage points.

**Results:**

The household survey identified poor access to quality seed (86.77% of respondents), climate-related crop losses (75.88%), unstable market prices (79.96%), and high fertilizer costs (47.67%) as the most prevalent constraints. The GMB process revealed these challenges as components of self-reinforcing feedback loops: a low-productivity trap in the seed system rooted in insufficient ISRA breeder seed capacity; a processor marginalization loop driven by Chinese trader dominance that diverts raw material away from SONACOS (operating at 35–40% capacity); and a cross-sectoral institutional misalignment trap in which subsidy capture, weak seed quality enforcement, and misaligned export policies operate as mutually reinforcing barriers.

**Discussion:**

Policy pathways emerging from GMB deliberations include strengthening ISRA’s breeder seed capacity; reforming fertilizer subsidy delivery through cooperatives; expanding scale-appropriate mechanization; integrating climate information services; establishing price stabilization mechanisms; and introducing dynamic export regulation to balance farmer income with domestic processor viability. Underpinning all recommendations is the need for a coordinated governance structure that addresses institutional misalignments and enables equitable, resilient, and sustainable growth across Senegal’s groundnut value chain.

## 1 Introduction

Groundnut value chain (GVC) plays a very important role in Senegal’s agricultural landscape and economy, covering about 1.25 million hectares, representing approximately 40% of the total cultivated area. Predominantly cultivated by smallholder farmers under drylands, it serves as a primary source of income and food security for a large proportion of the rural population (1, 2). The GVC plays a critical role in various economic activities, including input industries, processing, and exports. The GVC not only generates significant employment opportunities but also contributes significantly to income, nutrition, and livelihoods, particularly in the dryland regions of the country (3, 4). Groundnuts are a major contributor to the export earnings (5).

**FIGURE 1:**
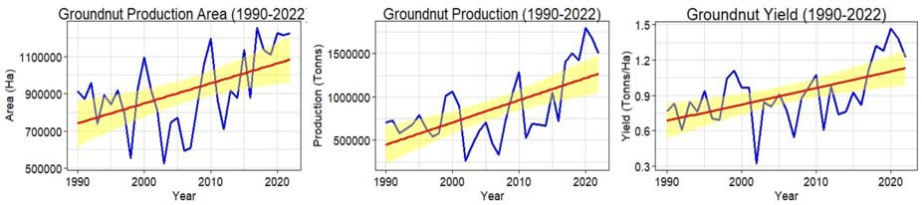
Groundnut area, production, and yield in Senegal (1990–2022); Data was sourced from the FAO.

Though it plays a vital role in the national economy, the GVC has been increasingly facing several challenges both on the demand and supply side (3, 4, 6). Its cropped area, yield, and production have remained unstable due to multiple factors (Figure 1). Climate change, outdated industry infrastructure, insufficient investment, inefficiencies in the value chain, and the unregulated entry of international traders, such as from China, have collectively posed significant risks, amplifying the vulnerability of the GVC. Despite collaborative efforts by different stakeholders, the GVC in Senegal remains underperforming and vulnerable.

Given these challenges, there is an urgent need to design and implement inclusive systemic solutions for enhancing the resilience of the groundnut value chain. Groundnut smallholder farmers, facing varied climate and market risks, require targeted interventions to enhance resilience. Moreover, the broader industry, specifically the groundnut oil sector, faces issues such as low-capacity utilization, increased competition, and diminishing profitability (7, 8).

In this study, resilience is defined as the capacity of the groundnut value chain, encompassing farmers, processors, traders, and supporting institutions, to absorb disturbances, adapt to changing conditions, and transform toward more inclusive and sustainable configurations in the face of recurrent shocks (9–11). Operationally, resilience is understood along three complementary dimensions: absorptive capacity (the ability of farmers and firms to cope with and recover from climate shocks, price volatility, and input disruptions without losing core functionality); adaptive capacity (the ability of actors and institutions to adjust practices, strategies, and organizational arrangements in response to emerging stresses); and transformative capacity (the ability of the system as a whole to restructure governance, market design, and institutional relationships when existing configurations are no longer viable) (9, 12, 13). Applied to Senegal’s groundnut value chain, resilience therefore encompasses the stability of smallholder farmer livelihoods under climate and market shocks, the operational viability of domestic processors under competitive pressure, and the adaptive and transformative capacity of the institutional framework governing seeds, subsidies, and trade.

In this complex scenario, adopting linear approaches proves insufficient for making effective policies to uplift such diverse value chains. The multifaceted nature of challenges demands a contextualized and comprehensive strategy to address the complexities of the groundnut value chain in Senegal (14, 15).

Value chains are inherently complex adaptive systems, characterized by non-linear interactions, feedback dynamics, and emergent behaviors that cannot be adequately captured through simple input-output frameworks (16–18). Scholars in the field of value chain analysis have increasingly recognized that complexity arises from multiple, interacting dimensions: the multiplicity of actors with divergent interests and power asymmetries, the embeddedness of transactions in institutional and socio-cultural contexts, the presence of feedback loops between supply and demand nodes, and the co-evolution of market and non-market governance structures (19, 20). In agri-food systems in particular, this complexity is further amplified by environmental variability, climate-induced uncertainty, and path-dependent technological and institutional configurations (21, 22). From a systems perspective, value chain complexity implies that interventions targeting a single node or actor may generate unintended consequences elsewhere in the system, a phenomenon well-documented in system dynamics and complexity theory literature (18, 23–25). Understanding value chain dynamics therefore demands approaches that can simultaneously account for structural interdependencies, actor heterogeneity, and the temporal evolution of system behavior (17, 26, 27, 28).

The theoretical framing of value chain complexity draws on several complementary bodies of literature. Global value chain theory, developed by Gereffi et al. (17), provides a governance-oriented lens through which to understand how power relations, lead firm strategies, and coordination mechanisms shape upgrading trajectories and distributional outcomes. Complementing this, the market systems development framework (29) directs attention to the systemic causes of market dysfunction, including weak supporting functions, distorted rules, and exclusionary norms, rather than isolated constraints. Meanwhile, system dynamics (23, 30) offers formal tools for conceptualizing and mapping the feedback structures that give rise to persistent system problems such as low productivity traps, price volatility, and institutional failures. Together, these frameworks suggest that complexity in agricultural value chains is not merely an empirical observation but a structural property of the system, rooted in the co-existence of multiple feedback loops, coordination failures, and interdependent actor strategies. A detailed description of the structure of the groundnut value chain in Senegal, including actors, functions, and institutional relationships, is provided in the Appendix.

Over the past decade, there has been a notable shift in the approach to value chain research, moving toward a market systems development perspective. Within this evolving framework, gaining a deeper understanding of the broader market system, considering different nodes of the value chain, becomes crucial for developing sustainable and equitable solutions for disadvantaged communities (31, 32). Concurrently, a significant facet of this transformation involves the application of system dynamics (SD) as an analytical tool. This tool is employed to comprehend and model the intricate interactions among social, economic, biological, and environmental factors that drive value chain dynamics. Additionally, it enables the quantification of the impacts of potential interventions (26, 33). Group Model Building involves the co-creation of system dynamics models with stakeholders, whose collective knowledge and expertise provide insights into structure and parameters associated with system phenomenon (34).

In this study, besides using primary and secondary sources of data and extensive focused discussions with key stakeholders of the value chain individually, we employed the participatory Group Model Building (GMB) approach^1^ to analyze the complexities of the GVC in Senegal. While existing literature addresses various aspects of the groundnut value chain in Senegal, this research stands out and adds value in terms of a comprehensive understanding of complexities and delineating policy perspectives through Group Model Building.

This study pursues three interlinked objectives. First, to map and analyze the dynamics of Senegal’s groundnut value chain using a participatory group model-building approach. Second, to identify key challenges and leverage points shaping productivity, resilience, and inclusiveness within the value chain. Third, to propose actionable policy pathways for strengthening the sustainability and competitiveness of the groundnut sector.

Prior studies on Senegal’s groundnut value chain have made important contributions in documenting actor structures, market linkages, and production constraints (3, 5, 6, 35). However, these studies have largely employed static, linear frameworks that identify constraints at individual nodes of the chain without capturing the feedback dynamics, institutional interdependencies, and system-level trade-offs that govern the chain’s behavior over time. The present study makes three distinct contributions that go beyond this existing body of work.

First, by applying GMB, this study reveals that the most critical challenges in the groundnut value chain, poor seed access, fertilizer shortfalls, and low farm profitability, are not independent bottlenecks but components of self-reinforcing feedback loops. For example, weak institutional capacity at ISRA constrains breeder seed supply, which reduces genetic gains and yields, which suppresses farmer income, which weakens demand signals for improved varieties, which in turn reinforces ISRA’s underfunding and institutional inertia. This reinforcing loop cannot be identified or broken through isolated interventions targeting any single node; it requires coordinated systemic action, an insight that only emerges through causal loop mapping.

Second, this study exposes a structural trade-off between export-led farmer income and domestic processor viability, and critically, reveals the unintended consequences of instinctive policy responses to this tension. The dominance of Chinese traders, who offer higher prices, creates a reinforcing loop of raw material diversion away from SONACOS, driving its capacity utilization down to 35%–40% and raising its unit costs, making it even less price-competitive. The GMB process revealed that a blanket export ban, the most commonly proposed policy response, would sever farmer income without guaranteeing SONACOS procurement efficiency, likely generating outcomes worse than the status quo. This policy paradox, and the dynamic export regulation approach proposed as an alternative, emerged specifically from the cross-actor causal mapping exercise and would not have been visible through conventional value chain analysis or stakeholder consultations alone (17, 26).

Third, GMB uniquely surfaced institutional misalignment as a system property rather than a series of isolated governance failures. The simultaneous operation of fertilizer subsidies largely appropriated by the political elites, seed quality standards unenforced by DISEM, and export policies misaligned with processor support, while individually documented in the literature, were revealed through the causal mapping process to operate as a mutually reinforcing institutional trap that systematically excludes smallholders and perpetuates system fragility. This cross-sectoral, cross-actor insight is the distinctive epistemic contribution of the GMB approach and establishes the methodological foundation for the policy pathways proposed in this study.

In the subsequent sections of this paper, we have discussed the method used for GMB. Following the method section, the results of analysis based on the value chain mapping and GMB workshop are presented in two subsections; the first is the basic orientation of the groundnut Industry and the second is on enabling policy perspective for the groundnut industry. In the next section, the results are discussed. Finally, the conclusions are presented.

## 2 Data and methods

This study uses both secondary and primary data sources to assess the present status and dynamics of groundnut value chain and the challenges faced by farmers in Senegal. The combination of these data sources allowed for a comprehensive analysis of the constraints and opportunities within the groundnut value chain identifying potential entry points to enhance resilience and performance of the groundnut value chain in Senegal.

### 2.1 Secondary data

Secondary data were obtained from the Food and Agriculture Organization (FAO) database to examine historical trends in groundnut area, production, and yield at the national level. These data provided an overview of long-term changes in groundnut cultivation and productivity, allowing for a contextual understanding of how the sector has evolved over time.

### 2.2 Study area and sampling approach

The farm household level data collection was undertaken within the intervention area of the Accelerating Impacts of CGIAR Climate Research for Africa (AICCRA) project in the Senegal’s “peanut basin” spanning 18 villages in the regions of Kaffrine, Louga, and Thiès. These regions were selected based on their significance in groundnut production, as they collectively represent key hubs of groundnut farming in Senegal. Additionally, these regions exhibit varying levels of market integration, climate vulnerabilities, and institutional support, allowing for an in-depth examination of the diverse challenges faced by groundnut farmers.

To structure the sampling approach, three central villages were first identified: Daga Birame in Kaffrine, Thiel in Louga, and Meouane in Thiès. These villages were selected due to their strategic location in key production zones and their active engagement in groundnut farming. The remaining 15 villages were chosen based on their proximity and relevance to these central locations, ensuring a comprehensive representation of groundnut-producing communities within the study area. While this purposive sampling approach maximizes contextual diversity and relevance for value chain analysis, it limits the generalizability of quantitative findings to the broader Senegalese groundnut farming population beyond the peanut basin. Readers should interpret quantitative findings accordingly.

### 2.3 Primary data collection

Primary data collection was carried out using three complementary methods applied at different levels of the value chain system: household level, community/village level, and institutional/actor level, providing both quantitative and qualitative insights into the groundnut value chain.

**a. Household Surveys**: The study leveraged baseline survey data collected in June 2022, structured questionnaires administered by trained enumerators through face-to-face interviews covering a total of 540 households across the 18 selected villages (30 households from each of the 18 villages). The survey gathered detailed information on household demographics, farm characteristics, production practices, input use, marketing strategies, access to services, and key constraints faced by groundnut farmers. However, in the final analysis we dropped 36 household due to incomplete information and used data from 503 households.

**b. Focus Group Discussions (FGDs)**: A total of 20 FGDs were conducted across 18 villages to gain an in-depth understanding of local farming practices, perceptions of value chain constraints, climate adaptation strategies, and community-level dynamics influencing production and marketing decisions. The FGDs included a diverse range of participants, including smallholder farmers, women’s groups, and cooperative members, ensuring the inclusion of multiple perspectives.

**c. Key Informant Interviews (KIIs)**: Semi-structured questionnaires were used as the guiding instrument, allowing flexibility to probe context-specific insights while ensuring coverage of core themes such as production practices, input access, market dynamics, and policy perspectives. To capture insights from various stakeholders in the groundnut value chain, key informant interviews were conducted with representatives from:

∘ The Société Nationale de Commercialization des Oléagineux du Sénégal (SONACOS), the primary entity responsible for groundnut processing and commercialization in Senegal.
∘ The Senegalese Institute of Agricultural Research (CERAAS-ISRA) provides insights on research and development efforts for groundnut improvement.
∘ Policymakers and high-level officials from the Ministry of Agriculture, offering perspectives on policy frameworks, regulations, and strategic interventions in the sector.
∘ Farmer cooperatives, traders, and processors, shedding light on the functioning of supply chains, market linkages, and challenges faced by different actors within the value chain.
∘ National Interprofessional Council for the Groundnut Sector (CNIA) – A multi-stakeholder committee playing a role in negotiating the groundnut grain price among others
∘ Réseau des Organizations Paysannes et Pastorales du Sénégal (RESOPP)- a network of farmers and pastoralists cooperatives
∘ Initiative Prospective Agricole et Rurale (IPAR)- a think tank working on agricultural value chains
∘ National Agency for Agricultural and Rural Council (ANCAR), which is responsible for promoting, facilitating, and managing agricultural and rural advisory services.
∘ ASPRODEB- Senegalese association for the promotion of development at the base.
∘ ANACIM- National Agency of Civil Aviation and Meteorology, which also provides weather forecasting services for the agriculture sector.
∘ Private sector entities engaged in climate information services
∘ National Union of Interprofessional Seed Producers (UNIS)

### 2.4 Group Model Building

During the last several years, considerable research and policy effort has sought to enhance the resilience of Senegal’s groundnut value chain. Yet, the system remains highly complex, shaped by interdependent nodes across input delivery, production, processing, and trade. Persistent problems such as weak seed systems, fragmented markets, volatile prices, and climate risks cannot be addressed through isolated interventions because they are rooted in feedback loops and institutional misalignments. A major source of difficulty is the diversity of actors involved, from smallholder farmers and cooperatives to processors, exporters, and government agencies, each pursuing distinct, and often competing, objectives. These divergent interests not only create coordination failures but also obscure the underlying dynamics that generate recurring vulnerabilities. In this context, it becomes essential to convene all critical stakeholders on a common platform, where collective insights can be used to uncover system linkages, negotiate trade-offs, and develop a shared understanding of pathways toward a sustainable and inclusive groundnut value chain. Against this backdrop, Group Model Building offered a structured platform to translate these diverse perspectives into a shared causal framework. It provided the methodological bridge from fragmented experiences to a collective systems view of the groundnut value chain (36, 37).

We utilized GMB to develop stakeholders’ common understanding of the underlying structures and relationships across different nodes of the groundnut value chain in Senegal. Before the GMB workshop, we conducted individual discussions with several key stakeholders of the value chain to understand their individual perspectives on status, constraints, opportunities, and policy needs (36). It is important to note that the GMB workshop did not operate in isolation from grassroots commercial realities. The causal structures explored during the workshop were systematically grounded in rich primary data collected directly from value chain operators prior to the GMB exercise: a baseline household survey of 503 smallholder farmers, 20 Focus Group Discussions held across 18 villages with farmers, women’s groups, and cooperative members, and individual Key Informant Interviews with farmers, traders, processors, input suppliers, and industry representatives. At the outset of each GMB session, findings from these primary data sources were presented as the empirical foundation for the causal mapping exercise, ensuring that the lived experiences and commercial perspectives of grassroots actors were explicitly embedded in the modeling process. The GMB workshop thus served as an integrative and interpretive layer built upon, rather than a substitute for, direct engagement with value chain operators (36, 38).

Group Model Building allows stakeholders to explore the system dynamics from an endogenous perspective, focusing on feedback loops (38, 39). The GMB process involves structured workshops designed to elicit variables and create increasingly refined qualitative diagrams representing the interconnectedness and feedback loops within the system (40–44). The workshop design is based on established sequences but is adapted by a core modeling team comprising modelers, subject matter experts, and value chain stakeholders, ensuring the inclusion of diverse perspectives (30, 39). The resulting qualitative models employed causal loop diagrams and stock and flow diagrams, effectively capturing the feedback mechanisms that underline the dynamic behavior of the system (30). A central tenet of system dynamics is that system behavior emerges via reinforcing and balancing feedback loops that promote equilibrium or disequilibrium in a system, depending on loop interactions and the strengths of loop inputs. A reinforcing loop denotes a mechanism that amplifies change in the system while a balancing loop counteracts change (45).

In March 2023, we conducted an exploratory GMB workshop at the ILRI office in Dakar, Senegal, followed by a subsequent exercise in mid-April 2023. The workshop aimed to capture dynamics within the GVC and identify potential scenarios for assessment in the value chain model. Participants included project facilitators, a reference group of various stakeholders, and individuals engaged in Group Model Building. The workshop comprised 14 participants, including representatives of farmers (2), traders (1), processors (1), government (2), think tanks and universities (3), and research for development institutions (5) (Table 1). Among the 14 participants, 4 were women.

**TABLE 1:** Group Model Building (GMB) workshop participants, roles, and primary contribution to model elements.

Participant category	*N*	Value chain node represented	Primary contribution to causal map
Farmers/farmer cooperative representatives	2	Production, input access	Supply-side constraints; seed and fertilizer access loops; climate risk variables
Trader	1	Marketing, trade	Price discovery mechanisms; export demand dynamics
Processor	1	Processing, industry	SONACOS capacity utilization loop; raw material procurement links
Government representatives	2	Policy, regulation	Subsidy delivery channels; export policy variables; regulatory levers
Think tanks/universities	3	Research, policy analysis	Cross-system feedback identification; institutional misalignment variables
R&D institutions	5	Research, technology	Seed system variables; climate information services; technology adoption loops
Total	14		
of whom women	4		

Each participant group contributed primarily to the causal variables and linkages within their domain of direct experience, while cross-cutting feedback loops particularly those connecting institutional policies to farmer-level outcomes were refined through plenary deliberation across all groups.

The GMB workshop was explicitly grounded in the primary data collected prior to the sessions. At the opening of each workshop day, the facilitation team presented a structured evidence brief to all participants, which included: (i) frequency distributions of key farmer-reported constraints from the 503-household baseline survey (e.g., 86.77% reporting poor seed access, 79.96% reporting price volatility, 75.88% reporting climate-related losses); (ii) thematic summaries of challenge clusters identified across the 20 FGDs; and (iii) a synthesis of institutional and market perspectives emerging from the KIIs. These findings served as the empirical starting point for variable identification in the causal mapping sessions – participants were asked to explain the causal mechanisms behind the patterns observed in the data, rather than simply listing perceived problems. This approach ensured that the GMB process moved from evidence to causation, not from opinion to opinion (36, 38). Variable identification followed a structured elicitation protocol. In the first session, participants were divided into actor-homogeneous sub-groups (farmers and cooperatives; traders and processors; government and research institutions) and asked independently to identify the key variables shaping groundnut value chain performance in their domain of experience. Sub-group outputs were then presented in plenary, where the facilitation team used affinity mapping to cluster overlapping variables and identify areas of consensus and divergence. Variables that appeared across multiple sub-groups were prioritized for inclusion in the causal loop diagrams. The facilitation team – comprising a system dynamics modeler, a value chain specialist, and a domain expert – made final decisions on variable inclusion based on three criteria: empirical grounding in the pre-workshop survey data, relevance to at least two value chain nodes, and causal tractability within a feedback loop structure (43, 44).

Once an initial causal map was constructed by the facilitation team based on plenary discussions, it was projected and presented to all participants for collective validation. Each proposed causal link was reviewed by asking participants to confirm, challenge, or modify the direction and polarity of the relationship. Participants were explicitly invited to indicate whether a causal connection was: (a) strong and well-evidenced – represented as a solid line in the diagram; (b) weak, poorly coordinated, or institutionally underdeveloped – represented as a dotted line; or (c) absent but desirable – flagged as a missing link requiring policy intervention. The dotted-line convention thus reflects a specific methodological judgment made collectively by participants: these are not missing data but structurally weak relationships that exist in the system but operate below their potential. This distinction was central to the analytical contribution of the GMB process and directly informed the policy prioritization in subsequent sessions (30, 40).

Divergent perspectives among participant groups – particularly between farmers and processors regarding export policy, and between government representatives and private sector actors regarding subsidy targeting – were managed through a structured deliberative process. When conflicting causal claims arose, the facilitation team used three strategies: first, the disagreement was explicitly named and recorded on a visible conflict log; second, participants were invited to present the evidence or experience underlying their position, drawing on the pre-workshop data briefs where relevant; and third, where consensus could not be reached, both perspectives were retained in the model as alternative causal pathways, with the contested link flagged for sensitivity analysis. This approach prevented dominant voices – typically institutional and research participants – from overriding grassroots commercial perspectives and ensured that the causal loop diagrams reflected genuine systemic complexity rather than consensus imposed by powerful actors (36, 38).

To assess the robustness of the resulting causal loop diagrams, two post-workshop validation steps were undertaken. First, the preliminary diagrams were circulated to KII participants who had not attended the workshop – including farmer cooperative leaders, SONACOS representatives, and Ministry of Agriculture officials – and their feedback was used to verify the accuracy of key causal relationships and the completeness of the variable set. Second, the facilitation team conducted an internal structural review in which each feedback loop was stress-tested by asking: “If this link were removed or reversed, would the system behavior described by other participants still hold?” Links that failed this test were either strengthened with additional evidence or reclassified as dotted lines. No formal quantitative sensitivity analysis was conducted at this qualitative stage, consistent with established GMB practice for qualitative causal loop mapping; however, quantitative simulation and sensitivity testing using platforms such as Vensim or Stella is recommended as a priority for future research (23, 33).

After initial causal mapping sessions, the team refined raw causal loop diagrams through a participatory iterative process. In the subsequent session, preliminary causal maps were presented to all participants for critique and real-time revision. The final session involved presenting the revised version to participants. Throughout these sessions, participants were encouraged to identify challenges at different nodes of the groundnut value chain, their interconnections potential feedback effect, and potential policy interventions to address the challenges and optimize value chain outcomes.

In addition to the GMB sessions, several Key Informant Interviews (KIIs) were conducted with the various major stakeholders of the GVC. The stakeholders included farmers’ organizations, groundnut industry associations, processors, farmers, agri-inputs dealers, NGOs, and researchers. These interviews provided background information and in-depth knowledge about various actors and processes, interconnections, and addressing different aspects of the groundnut value chain in Senegal.

A methodological limitation of the GMB workshop design should be acknowledged. Of the 14 workshop participants, only 4 were direct value chain operators (2 farmers, 1 trader, 1 processor), while the remaining 10 represented institutional, government, academic, and research constituencies. This composition reflects a deliberate design choice consistent with established GMB practice in complex agri-food systems, where workshops are structured to map system-level feedback structures and policy levers rather than to proportionally represent actor numbers (36, 43). Nonetheless, this skewed composition may have introduced an institutional bias in the interpretation of feedback dynamics. To mitigate this risk, the workshop outputs were validated against the extensive primary data collected from farmers and commercial actors, and the preliminary causal loop diagrams were presented back to participants, including grassroots representatives, for critique and real-time revision. Future applications of GMB in similar value chain contexts should aim for a more balanced actor representation to strengthen the grassroots legitimacy of the resulting causal maps.

## 3 Results and discussions

**FIGURE 2:**
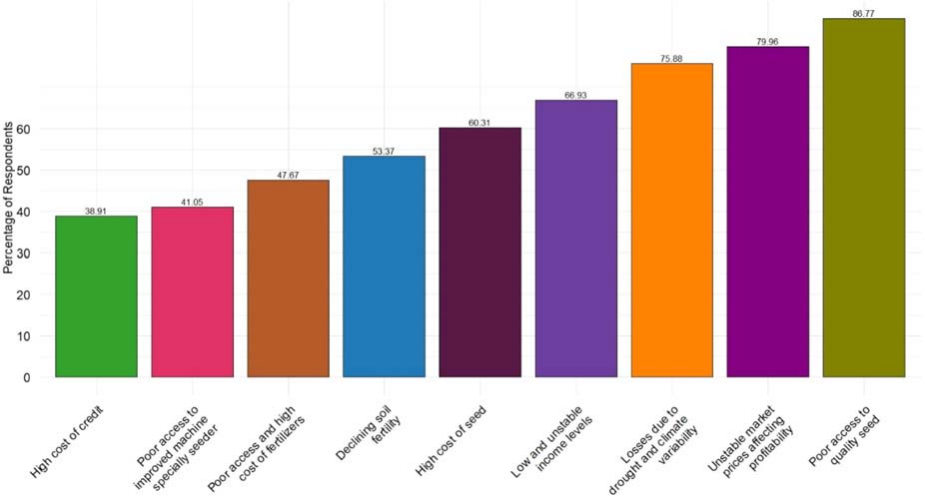
Challenges faced by the groundnut farmers in Senegal.

The groundnut value chain in Senegal^2^ was found to be in a very complex setting. Based on our understanding of various aspects of the groundnut value chain gained through farm household level study, we further engaged with and discussed its various aspects with different value chain stakeholders for a greater understanding of the nature of constraints and opportunities. In our farm household-level study results, we identified several critical challenges faced by groundnut farmers in Senegal, which impacted groundnut productivity, profitability, and overall resilience (Figure 2).

Before presenting the findings, it is important to clarify what the GMB approach contributed beyond what the household survey, FGDs, and KIIs alone could have established. The large-scale primary data collection identified the prevalence and magnitude of individual constraints across the value chain. The GMB workshop then went further: it enabled diverse stakeholders to collectively trace the causal connections between these constraints, identify feedback loops that perpetuate system dysfunction, surface institutional misalignments that no single actor could perceive from their own vantage point, and negotiate the trade-offs embedded in proposed policy responses. The three system-level insights that emerged, a reinforcing low-productivity trap in the seed system, a structural export-processing trade-off with paradoxical policy implications, and a cross-sectoral institutional misalignment trap, are the specific products of this participatory systems lens and structure the analysis presented below (23, 36, 38).

### 3.1 Seed-related challenges

The most prominent issue for farmers was poor access to quality seed of resilient and high-yielding varieties, reported by 86.77% of respondents. This indicates a significant bottleneck in achieving good and stable yields and ensuring improved varieties are accessible to farmers. The high cost of seed (60.31%) further exacerbated this challenge, limiting farmers’ ability to invest in better inputs.

### 3.2 Market and financial constraints

Market-related challenges were also severe, with unstable market prices affecting profitability being a major concern for 79.96% of farmers. The volatility in prices creates uncertainty and limits farmers’ income stability. Additionally, low- and unstable-income levels (66.93%) further reflect the economic vulnerability of groundnut producers. The high cost of credit (38.91%) was another barrier that restrict farmers’ ability to invest in improved practices and inputs.

### 3.3 Climate and environmental factors

Losses due to drought and climate variability were the major constraints reported by 75.88% of respondents, highlighting the severe impact of climate change on groundnut production in Senegal. Declining soil fertility (53.3%), attributed to both long-term climatic shifts and unsustainable soil management practices, further exacerbates the environmental challenges. In farmers’ perceptions, the drought, strong winds, and soil fertility decline caused by climate change were the main climate hazards that adversely affected groundnut crop production (46).

### 3.4 Input and mechanization challenges

Farmers also faced high production costs, with high fertilizer costs (47.67%) being a significant concern. Limited access to affordable fertilizers leads to reduced yields and overall farm productivity. Additionally, poor access to improved machinery, especially groundnut seeders (for 41.05% farmers), hindered the efficiency of sowing operations, potentially affecting planting times, net sown area, and yields.

Building on the farm household-level analysis, we further discussed these insights with different stakeholders in the GMB workshops. Based on these discussions during the GMB workshops we have divided this section into two subsections: (a) Understanding the functioning of GVC in Senegal and (b) Potential policy pathways for enhancing the resilience of the groundnut value chain in Senegal.

**A. Understanding the functioning of the groundnut value chain in Senegal**

To comprehensively analyze the complex dynamics of the groundnut industry in Senegal, a detailed examination of both the supply and demand sides, along with the underlying drivers of the production system and price discovery processes, was examined.

### 3.5 Supply side

The supply side of Senegal’s groundnut value chain is shaped by a deeply interconnected system of input delivery mechanisms, environmental risk exposure, production dynamics, and institutional relationships. At its core lies the farmer’s capacity to access and effectively use critical inputs and improved technologies, seeds, fertilizers, mechanization, and timely agronomic advisories. Yet these channels are often fragmented, undercoordinated, and underperforming. As indicated by the causal loop analysis, several of these channels represent structurally weak or thin linkages, dotted lines in the system architecture, that require strategic strengthening to unlock productivity and resilience gains.

**FIGURE 3:**
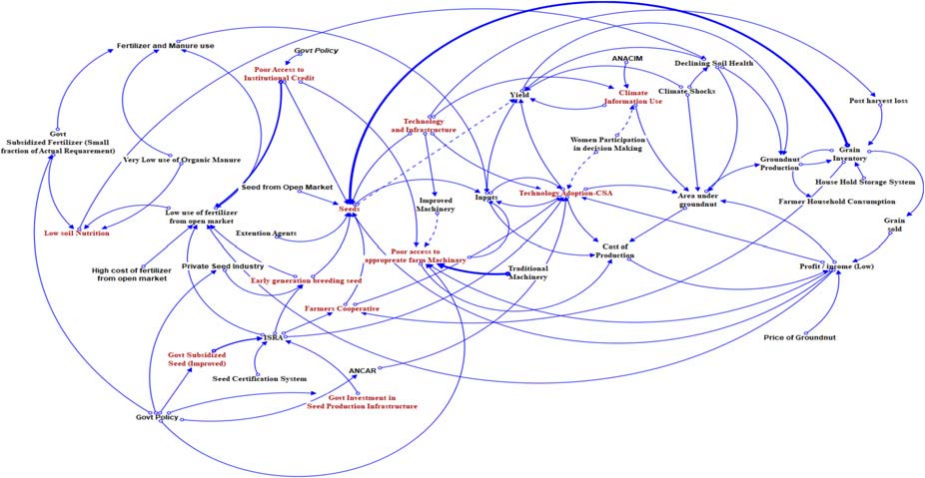
Supply side causal loop diagram of the groundnut value chain in Senegal. In the diagram, red-labeled nodes indicate constrained or underperforming variables; dotted lines represent weak, poorly coordinated, or underdeveloped causal links requiring policy attention; and solid lines represent active causal relationships. Arrows indicate the direction of causal influence.

Access to seed remains one of the most fundamental bottlenecks in the system. While multiple acquisition channels exist, self-saved seed, the open market, cooperatives, and government-subsidized schemes, only about 20% of farmers reportedly have access to good-quality seeds (47). The Senegalese Agricultural Research Institute (ISRA), which serves as the sole producer of breeder seed, has insufficient capacity and infrastructure to meet national demand. The linkage between ISRA and downstream multipliers (cooperatives, certified seed producers) is a critical dotted-line connection: weak, poorly coordinated, and lacking financial and logistical support. This results in limited varietal turnover, low genetic gains, and continued reliance on informal, lower-quality seed sources, reinforcing a cycle of low productivity. This constitutes a self-reinforcing low-productivity loop in the seed system: weak ISRA capacity constrains breeder seed supply, which reduces genetic gains and yields, which suppresses farmer income, which weakens demand signals for improved varieties, which in turn reinforces ISRA’s institutional underfunding, a closed reinforcing dynamic visible in Figure 3.

Fertilizer access presents a parallel constraint. Although the state offers subsidies on inorganic fertilizers, only a small proportion of farmers get enough, and the majority of farmers receive only 5–10 kg of subsidized fertilizer per season, which is far below the agronomic recommendations. Delivery of subsidized fertilizer is plagued by targeting inefficiencies, allocation delays, and distribution bottlenecks. The dotted-line weakness here lies in the delivery chain between centralized government programs and actual on-farm application. Trust in the subsidy system erodes when access is inconsistent, especially among smallholders and women farmers, leading to underinvestment in soil fertility. Further, very high prices of the fertilizers in the open market suppress their use, contributing to the chronic soil fertility challenge. This constitutes a parallel reinforcing dynamic in the input delivery system: inconsistent subsidy delivery erodes farmer trust, which reduces input investment, which lowers yields and income, which weakens the political case for subsidy reform, perpetuating the same targeting failures.

Mechanization was another high leverage but underdeveloped area. Most farmers rely on animal-drawn traditional seeders or tools, risk of missing the narrow sowing windows that are increasingly dictated by erratic rainfall. Mechanization subsidies exist but often fail to reach smallholders due to scale mismatches, lack of awareness, and limited financial access. The weak link between technology developers, service providers, and end-users, particularly small-scale and women farmers, is a crucial dotted-line dynamic. Strengthening this connection through scale-appropriate tools, community-based rental models, and cooperative-level aggregation could substantially increase land use efficiency and reduce labor bottlenecks.

Climate risks cut across all input systems, amplifying existing structural weaknesses. Delayed rains, heatwaves, and extended dry spells increase the importance of timely sowing and adaptive crop management (48, 49). While Senegal has a relatively strong capacity to generate climate information, the last-mile dissemination of Climate Information Services (CIS) remains weak, particularly for women farmers, who are often excluded from decision-making. This gendered information asymmetry is another critical dotted line, one that hinders adoption of Climate-Smart Agricultural Practices (CSAPs) and slows the system’s adaptive response to environmental stressors.

Gender dynamics were explicitly surfaced during the GMB workshop and are reflected in the supply-side causal loop diagram, in which “Women Participation in Decision Making” appears as a distinct variable linked to technology adoption and input use (Figure 3). This reflects stakeholder recognition that women’s limited participation in agricultural decision-making constrains technology adoption, input investment, and ultimately yields, a causal pathway consistent with the broader literature on gender and agricultural productivity in West Africa (50, 51). However, the household survey was not designed to generate gender-disaggregated data at the level of detail required to fully parameterize or analytically develop this causal pathway within the model. A more comprehensive integration of gendered feedback dynamics, including for example the loop linking information access to technology adoption, income, and women’s agency, is therefore beyond the scope of the present study and is identified as a priority for future research.

Post-harvest handling is equally affected by system fragmentation. Harvesting is delayed not only due to labor shortages but also due to limited access to appropriate machinery. Drying, sorting, and storage conditions are often substandard, resulting in post-harvest losses and potential aflatoxin risk (52). The interface between farm-level production and post-harvest support systems, including, extension services, storage infrastructure, and aggregation nodes, is a classic dotted-line weakness. Poor post-harvest handling not only reduces marketed volumes and quality but also undermines food safety and future seed viability, creating a reinforcing feedback loop that begins anew each planting season.

Institutional and organizational linkages further shape the performance of the supply system. Many farmers, especially women and resource-poor farmers, remain outside cooperative structures due to social, informational, and financial barriers. These exclusions weaken the channel through which farmers could otherwise access bundled services, input subsidies, credit, and training. Where cooperatives are functional, their role in seed production, machinery rental, and information dissemination proves transformative. Where absent, systemic inertia dominates. Strengthening cooperative governance, scale, and outreach emerges as a key entry point to transform these dotted lines into robust conduits of support and innovation.

Crucially, the economic viability of input investments remains fragile. Low and unstable profitability, caused by inconsistent yields, high input costs, post-harvest inefficiencies and low prices, discourages adoption of new technologies. This creates a negative feedback loop where weak returns deter reinvestment, reinforcing the very constraints that limit system performance.

In sum, the supply side of the groundnut value chain in Senegal was not only constrained due to poor access to appropriate technologies, or incoherent policies, but by a constellation of strategically weak, under-connected, or under-leveraged system relationships, the dotted lines in the causal structure. These include broken or thin linkages between seed institutions and cooperatives, weak last-mile delivery of subsidies and weather-based contextualized agro-advisories, insufficient access to mechanization, and low organizational inclusion of marginalized groups. Addressing these bottlenecks through targeted interventions, governance reforms, and institutional innovations would be key to enhancing productivity, inclusiveness, and resilience across the groundnut system. In resilience terms, these dotted-line weaknesses represent failures of absorptive capacity, leaving farmers unable to buffer against seed shortfalls, fertilizer shortages, and climate shocks, and of adaptive capacity, as fragmented institutional channels prevent timely adjustment of input delivery and agronomic advice in response to changing conditions (13).

### 3.6 Demand side

**FIGURE 4:**
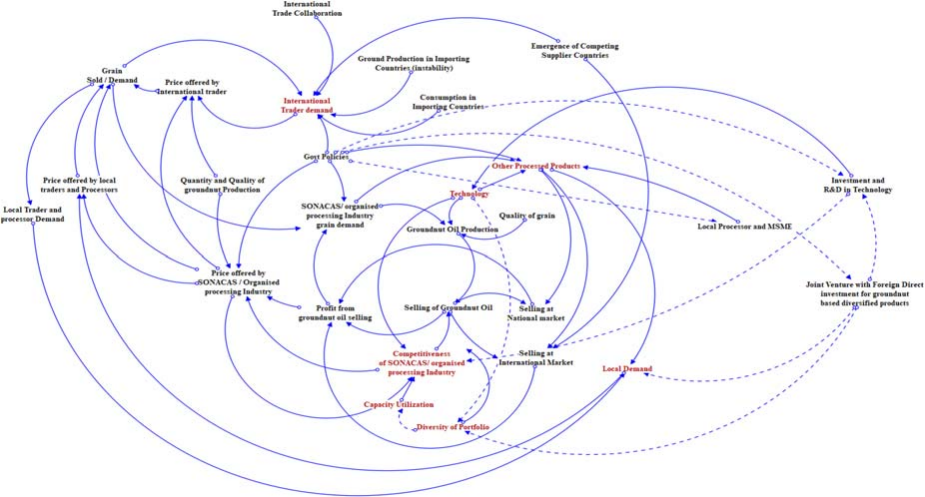
Demand side causal loop diagram of the groundnut value chain in Senegal. In the diagram, red-labeled nodes indicate constrained or underperforming variables; dotted lines represent weak, poorly coordinated, or underdeveloped causal links requiring policy attention; and solid lines represent active causal relationships. Arrows indicate the direction of causal influence.

The demand side of Senegal’s groundnut value chain (Figure 4) is mainly structured around three interrelated market channels: international traders, domestic formal processors such as SONACAS, and a distributed network of local traders and artisanal processors. Each of these channels reflects distinct procurement models, value creation logics, and institutional relationships. While they appear as parallel markets, but, they interact dynamically, often reinforcing imbalances, price volatility, and underutilized capacity across the system. Critically, as illustrated in the causal loop analysis, several of the most important challenges stem not only from constrained nodes of the value chain represented in red color, but also from the underdeveloped or thin relationships, represented by dotted-line channels, that require strategic strengthening.

At the apex were international traders, predominantly from China and Vietnam, who have dominated Senegal’s groundnut grain export landscape in recent years. Over the past decade, China alone has accounted for more than 90% of groundnut grain exports (11). These actors engage farmers directly through their local agents, offering marginally higher prices and quicker payments than domestic processors, but often target better quality grains. This creates a strong pull toward informal transactions, bypassing formal systems of quality assurance, traceability, and regulation. The appeal of these buyers is amplified by their low transaction costs and minimal post-harvest requirements. However, the institutional connections between these traders and the broader value chain, particularly in terms of quality control, long-term contracts, and market predictability, remain weak. This dotted-line relationship leaves farmers exposed to external volatility and weakens incentives to invest in storage, sorting, and aflatoxin control.

The dominance of export demand creates a reinforcing loop: higher prices attract more grain away from domestic processors, reducing their capacity utilization and long-term viability. But this structure is inherently fragile. International demand is volatile and subject to sudden disruptions, whether through phytosanitary regulations, trade restrictions, or market shocks. As experience shows that abrupt declines in export purchases can leave producers vulnerable without fallback options, particularly when domestic alternatives are underdeveloped.

Domestic formal processors, particularly SONACAS, occupy a structurally constrained position. Though historically central to Senegal’s groundnut economy, SONACAS now operates under multiple pressures: declining access to raw material, low-capacity utilization (estimated at 35%–40%), high unit processing costs, and a narrow product portfolio focused largely on oil and meal. The system suffers from a series of dotted-line weaknesses, including underdeveloped procurement linkages with farmer cooperatives and limited outreach mechanisms. Unlike international buyers who maintain active agent networks, SONACAS struggles with delayed payments, bureaucratic processes, and limited trust among producers. As a result, it offers less attractive prices and is often unable to compete for high-quality grain during the harvest season. This constitutes a reinforcing processor marginalization dynamic visible in Figure 4: export price premium diverts raw material away from SONACOS, reducing capacity utilization, raising unit costs, and further weakening SONACOS price competitiveness, deepening the very raw material shortage that initiated the loop.

These constraints create a negative feedback loop: limited supply reduces capacity utilization, which increases costs and reduces margins, further weakening SONACAS’s competitiveness. Compounding this is a lack of diversification of the product portfolio. The link between SONACAS and new product markets, such as snack foods, cosmetics, or fortified products and protein supplements, is currently thin and represents another dotted-line channel in need of investment and policy support. Without broader product offerings, SONACAS remains vulnerable to shifts in oil markets and cannot fully capitalize on the value potential of groundnuts.

The third demand node, comprising local traders and small-scale processors, plays a stabilizing but under-leveraged role. These actors are deeply embedded in rural economies and serve markets for snacks, informal retail, and direct household use. Their key advantage lies in immediacy: fast cash payments, minimal quality requirements, and flexible transactions. For many farmers, particularly in remote areas, they serve as the default buyers. However, their integration into formal quality systems, aggregation hubs, and upgrading programs remains limited, another dotted-line weakness. Lack of access to credit, safe storage, or packaging innovation, these actors are unable to scale or meet rising consumer expectations, both locally and abroad.

A further systemic constraint lies in the weak transmission of quality signals across the value chain. At present, neither international traders nor most local buyers rewards farmers for improved post-harvest practices or seed purity. This breaks the potential feedback loop between better quality and higher prices. The absence of functioning grading systems, reliable testing, and contract enforcement contributes to a flat pricing structure that fails to incentivize value-enhancing behaviors at the farm level. Strengthening this quality-price linkage, through aggregation centers, mobile testing, or structured marketing platforms, is a critical missing channel.

Finally, the policy interface between export regulation and domestic industry support remains contested. While SONACAS and other processors have called for heavy restrictions on groundnut exports to protect raw material access, however, a blanket bans risk suppressing prices and destabilizing farmer income if not accompanied by guaranteed procurement, price transparency, and efficient logistics. This misalignment between policy design and market functionality is another weak institutional channel that requires recalibration. Rather than blunt controls, dynamic export policies, such as registration systems, seasonal quotas, and incentive-based procurement guarantees, could better balance the interests of farmers and processors.

In sum, the demand system in Senegal’s groundnut value chain is not only defined solely by competing buyers, but by the strength, or weakness, of the relationships connecting them to producers and to each other. Many of the most powerful leverage points lie in the dotted lines: thin procurement links between SONACAS and cooperatives, weak integration of local processors into quality-based systems, absent incentives for post-harvest improvement, and fragile regulatory-market coordination. Strengthening these systemic weak ties will be essential to shift the groundnut value chain toward greater resilience, inclusiveness, and long-term competitiveness. In resilience terms, the dominance of volatile export demand and the structural weakness of SONACOS represent failures of transformative capacity the system, the institutional and market architecture needed to reconfigure itself toward stable, diversified, and domestically anchored demand structures (9, 10).

The policy pathways prioritize five leverage nodes, ISRA seed capacity, fertilizer subsidy delivery, cooperative strengthening, SONACOS procurement and diversification, and dynamic export regulation, because these nodes are each central to at least one reinforcing feedback loop identified in the GMB process, and intervening at these points offers the greatest potential to disrupt self-perpetuating system traps rather than addressing symptoms at peripheral nodes.

**B. Policy pathways for building resilience in the groundnut value chain**

The policy recommendations presented in this section emerged directly from GMB workshop deliberations, in which diverse stakeholders, government representatives, R&D institutions, processors, traders, and farmers, collectively identified, debated, and prioritized interventions based on their position within the causal loop structure of the value chain. Their comprehensiveness reflects the multi-actor scope of the GMB process rather than an exhaustive development agenda constructed by the authors, with feasibility, stakeholder trade-offs, and potential unintended consequences having been negotiated during the causal mapping sessions themselves. The recommendations are organized around both, the five high-leverage nodes identified through the GMB process, and three resilience capacities: interventions targeting seed system reform, fertilizer access, and mechanization primarily strengthen absorptive capacity; interventions targeting climate information services, cooperative strengthening, and market price stabilization primarily build adaptive capacity; and interventions targeting governance reform, institutional coordination, processing industry restructuring, and dynamic export regulation primarily build transformative capacity, enabling the system to reconfigure itself toward a more equitable and competitive long-term equilibrium (9, 10, 13).

**a. Access to seed**

Despite groundnut covering the largest cropped area of about 1.3 million hectares in Senegal, only about one-fourth of groundnut farmers have access to certified seeds (47). The lack of quality seeds of resilient, high-yielding varieties adversely affects production performance, creating a significant gap that needs urgent attention. The non-availability of a sufficient quantity of breeder or early-generation seed, as well as the lack of capacity of the public agencies to produce enough certified seeds of high-yielding and resilient varieties, are key constraints. These demand and supply gaps also have implications for its market price.

At present, the Institut Sénégalais de Recherche Agricole (ISRA) is the sole institution responsible for producing breeder seed in Senegal. However, its existing capacity and infrastructure are not sufficient and require substantial upgrades to meet the increasing demand for breeder seed. This is critical to ensure a consistent and sufficient supply of breeder seed to support the seed system.

Despite efforts to introduce short-duration seeds, such as cv. Sunugal, their acceptance among farmers has been limited, with a preference for older varieties that produce more fodder. Although improved groundnut varieties have been available since 2015, the shortage of certified seeds persists. A potential solution involves supporting local initiatives of seed production by engaging farmers’ cooperatives and giving preference to those with a proven track record of successful production.

The following policy recommendations emerged during the group model-building workshop:

- Given the potential demand for breeder seed, proper coordination, capacity building, and additional financial support must be ensured to achieve the government’s target of increasing breeder seed production by 40% (National Plan for Food Security 2024–2028).
- Implement a robust demand assessment and distribution system for breeder seeds. Conduct regular assessments to understand seed demand and formulate policies promoting liberalization of the seed sector to encourage cooperatives and private sector participation in variety development, seed production, and distribution.
- Policies to secure adequate financial resources for seed production, considering the critical role of the government in financing. Explore partnerships with non-government entities or organizations, as the collaboration between ISRA and the National Seed Trade Association (UNIS) agreed in 2021.
- Encourage farmers to join cooperatives for improved access to technology, inputs, markets, and government-supported programs. Support and encourage partnerships between cooperatives, e.g., Réseau Des Organizations Paysannes Et Pastorales Du Sénégal (RESOPP) and financial institutions, ensuring coverage for both members and non-members enabling access to credit to support technology adoption.
- Promote synergy and consortium-style collaboration among seed value chain actors, including UNIS, ASPRODEB, other farmers’ organizations, and relevant stakeholders. This cooperative approach will enhance the overall efficiency of the groundnut seed value chain. Safe storage of seeds at the farmers’ level is also important, the Purdue Improved Crop Storage (PICS) bags could be an affordable solution.
- There is an urgent need to revitalize ISRA’s capacity and infrastructure. Strategic investments and partnerships may be pursued to enhance the institution’s capabilities in seed production. Simultaneously, incentivizing private sector participation in seed production would be critical to the overall strengthening of the seed system, ensuring its resilience and sustainability.

**b. Access to modern inputs and associated subsidies**

The government allocates a significant budget toward subsidies to farmers for fertilizers, seeds, etc. However, a large proportion of farmers experience challenges due to insufficient availability and the poor targeting of subsidized fertilizers and improved seeds. As a result, many farmers, especially relatively resource-poor farmers, receive limited benefits of subsidies that lead to low use of fertilizers and improved seeds, consequently, low and unstable crop yields. Current subsidies fall short of meeting farmers’ needs; our household-level study found that, on average, each farmer received around 5 kg of subsidized fertilizer, which is a small fraction of the actual requirement, severely limiting agricultural productivity.

In addition, smallholder farmers encountered difficulties accessing appropriate machinery for small farms such as animal-drawn improved groundnut seeders, resulting in a reduction in area sown and lower productivity. Despite government provision for machinery subsidies, their reach remains limited, the lack of scale-appropriate mechanization exacerbating the challenges faced by smallholder farmers. Another critical concern was the exclusion of a significant proportion of farmers from cooperative structures. This exclusion hampered the potential benefits that cooperatives can provide in terms of scale, access to resources, technologies, and market opportunities.

The following policy recommendations emerged during the group model-building workshop.

- Implement a more inclusive and transparent mechanism for the delivery of subsidized inputs, incorporating an effective tracking system to ensure equitable distribution and prevent overcompensation. Efforts should focus on extending subsidies to reach excluded poor farmers who are currently underserved.
- Consider distributing subsidized fertilizers through farmers’ cooperatives to improve resource use efficiency. Simultaneously, encourage and build the capacity of farmers’ organizations/cooperatives on improved seed production and distribution, improving the overall quality and availability of seeds in the market. Aligning these efforts with government-supported programs to encourage broader engagement among farmers may be considered.
- Expand the reach of government schemes on farm machinery to better serve smallholders. Strengthening farmers’ cooperatives and organizations can facilitate collective access to machinery and optimize its use. Additionally, ensure effective tracking of subsidies to guarantee intended beneficiaries receive the necessary support.

**c. Enhancing Climate Resilience in Groundnut Production**

The increased frequency of climate extremes, heavy winds and unpredictable rainfall patterns has emerged as a substantial threat to groundnut production, leading to crop losses and reduced yields (49, 53). Senegal’s groundnut production also faces a critical issue marked by high production costs on account of limitations in seed, fertilizer, and machinery, inhibiting optimal use of inputs and technologies. The increasing risk of climate change and the frequency of crop failures have further eroded the profitability of the crop. This multifaceted challenge encompasses several key aspects that significantly impact the overall sustainability, resilience, and profitability of the agricultural sector. Limited access to climate-resilient improved agricultural technologies compounds the predicament, hindering the efficiency and productivity potential and increasing the risk in farming practices. The weakness in farmers’ cooperatives adds another layer to the challenge. Furthermore, the issue extends to the insufficient empowerment of women who play a crucial role in agricultural value chains.

The following policy recommendations emerged during the group model-building workshop.

- Implement strategies to boost crop yields through advanced farming practices, improved crop varieties, and efficient soil and water management.
- Enhance access to CSA technologies through training programs and facilitating affordable access to modern farming tools and inputs.
- Equip farmers with context-specific climate information, including early warning systems and climate-resilient farming practices tailored to local conditions. It would be critical to identify and support sustainable business models for climate information services (CIS). The CIS also needs to target other stakeholders of the value chain, such as seed and fertilizer suppliers, processors, traders, etc.
- Invest in strengthening farmers’ cooperatives and organizations to enhance collective bargaining power, resource sharing, and access to markets and technologies.
- Implement initiatives to empower women in agriculture by enabling their participation in farm decision-making and ensuring equitable access to resources, training, and technologies. Institutional innovations facilitating women collectives/groups to undertake the production, processing, and marketing of groundnut and other commodities may be an effective pathway.
- Tap into irrigation’s potential to mitigate water scarcity impacts and enhance agricultural productivity and resilience. Support the development of small irrigation infrastructure and rainwater conservation practices at the farm and landscape scales.
- Besides increased R and D investments, the research and extension services need to adopt a convergence approach to achieve large-scale adoption of resilient agricultural technologies and practices. Need-based promotion of precision farming and mechanization can enhance overall competitiveness.
- Diversification of cropping and farming systems is an important strategy to improve resilience and competitiveness.

**d. Mitigating Market Price Fluctuations**

The groundnut market experiences significant price fluctuations, impacting the income and financial stability of farmers and other value chain participants (54). The multiplicity of actors on the demand side, especially the unregulated international buyers (China, Vietnam, etc.), has resulted in concerns from the local industry.

- Establish a price stabilization mechanism, such as a stabilization fund, to reduce the impact of market volatility. This policy intervention can stabilize farmers’ incomes and encourage long-term investments in the sector.
- To address the concern of the local industry and have transparency, there could be a policy that would require any international buyer to register with the designated government agency before the crop harvest and indicate the potential amount of groundnuts to be purchased.

**e. Reducing Fertilizer Costs**

The high cost of fertilizers is a significant barrier for farmers, impacting their ability to invest in inputs and ultimately reducing yields.

- Negotiate with fertilizer suppliers to reduce costs, explore subsidy options, or consider bulk purchasing agreements to make fertilizers more affordable for farmers. Reducing the subsidy per unit of fertilizers but increasing the reach of farmers may be more rewarding for farmers and food and livelihood security.
- Engage cooperatives and extension systems to promote production and use of organic manure. Demonstrations, capacity building, awareness programs and policy incentives can help in increased integration of organic manure for soil health and resilience.

**f. Optimizing Groundnut Processing and Enhancing Competitiveness**

SONACOS, a key player in the groundnut value chain, is operating far below its capacity. At present, only about 35%–40% of its processing capacity is being utilized. Groundnut oil is the industry’s major product; however, there are limitations in its export market due to several factors. These factors lead to high transaction costs, inefficiencies in processing and distribution, and finally, affect the industry’s competitiveness.

- Need for a comprehensive assessment of the groundnut processing industry, its capacities, infrastructure and technology, product portfolio, market, and support strategies to optimize its operations. This may involve modernizing facilities, improving efficiency, and exploring new market opportunities for groundnut products in domestic and international markets.
- Develop and implement strategies to enhance the competitiveness of Senegalese groundnut oil, including obtaining quality certifications, investing in branding, and initiating targeted marketing campaigns to highlight its unique qualities.
- Policy support is needed for strengthening the micro, small, and medium enterprises including women groups involved in small-scale processing of groundnuts.
- Encourage diversification in processing by supporting research and development as well as joint ventures for new groundnut-based products. Provide incentives for processing facilities to explore and produce a broader range of value-added items.

**g. Harmonizing Policy Goals for Groundnut Value Chain**

Conflicting policy goals, such as providing subsidies while controlling prices, create inconsistencies and inefficiencies in the overall policy framework.

- Develop a coherent policy framework through stakeholder consultations and a comprehensive review of existing policies. This should balance the need for subsidies with price control objectives to create a consistent and effective policy environment.
- The need for an enabling policy environment ensuring access to innovations and technologies, financing, and markets as well as developing local capacity critical for building resilience to the value chain.
- Since the groundnut value chain has high social and political significance, the policies must align with national development goals and garner political support for successful implementation, while still prioritizing economic sustainability. Encourage transparency and evidence-based decision-making in policy processes.
- Need policies focused on bridging yield gaps, and improving income, including fair pricing mechanisms, efficiency improvements in the value chain, and financial support programs for both farmers and processors.

**h. Improving Coordination among Stakeholders**

The groundnut value chain involves multiple stakeholders and ministries, leading to coordination challenges and potential inefficiencies.

- Establish a coordinated governance structure involving all relevant stakeholders and ministries to streamline decision-making processes. Foster regular dialogue to address concerns and ensure effective implementation of policies.
- The value chain needs to be examined holistically, and policy actions designed to address the concerns of all stakeholders, farmers, processors, traders, input suppliers, and R and D agencies.

## 4 Conclusion

The groundnut value chain in Senegal exemplifies the tensions and opportunities that define agri-food systems in the context of climate change, market globalization, and structural transformation. This study reveals that the challenges are not merely technical or logistical but deeply embedded in systemic interactions, spanning institutional coordination, incentive structures, and socio-economic inequities. The findings underscore the importance of recognizing the groundnut sector as a dynamic system where feedback loops, actor behavior, and enabling conditions jointly shape outcomes.

The participatory group model-building approach has demonstrated its strength in making these dynamics visible to stakeholders, allowing for a shared diagnosis of problems and a common vision for future pathways. It moves beyond linear problem-solving toward a strategic framework for systemic adaptation, where resilience is not only about reducing vulnerability but also about enhancing the capacity of the system to innovate, reorganize, and evolve in response to internal and external shocks.

This system’s view also draws attention to critical yet often overlooked dimensions, such as the role of trust in institutions, the fragmentation of actor networks, and the long-term trade-offs between short-term relief measures and structural competitiveness. Building resilience, therefore, involves a dual imperative: stabilizing the current system while enabling transformation toward more inclusive, climate-adaptive, and value-creating futures.

Ultimately, the groundnut value chain in Senegal offers a microcosm of broader development challenges facing dryland agricultural economies. Its future will depend not only on targeted investments or technical fixes, but also on the capacity to orchestrate collective action, bridge scales of decision-making, and institutionalize learning. The qualitative causal loop diagrams developed through the GMB process represent a necessary first step toward a fully quantified system dynamics model. Future research should prioritize the empirical parameterization and simulation of these causal structures using dedicated system dynamics platforms such as Stella Architect or Vensim, which would enable rigorous scenario testing, sensitivity analysis, and quantitative impact assessment of proposed policy interventions (23, 33). This calls for an intentional reimagining of governance, market design, and policy coherence anchored in evidence, driven by collaboration, and responsive to the needs and aspirations of those at the heart of the system.

## Data Availability

The raw data supporting the conclusions of this article will be made available by the authors, without undue reservation.

## Footnotes

1 Group Model-Building involves a system dynamics model-building process wherein a client group actively participates in constructing the model. Typically, a gathering of around nine to fifteen individuals takes place in one or more sessions, guided by a modeling team, to collaboratively build and analyze the model.

2 Detailed groundnut value chain mapping has been presented in Appendix A.

## Appendix A | Groundnut Value Chain in Senegal.

Groundnut has long been a cornerstone of Senegal’s rural economy, linking thousands of smallholder farmers to national and international markets. The value chain encompasses a wide range of actors, from producers and input suppliers to traders, processors, exporters, and domestic consumers, each shaping the opportunities and challenges of the sector (Appendix Figure 1). Understanding how these actors interact reveals both the strengths of Senegal’s groundnut economy and the structural weaknesses that continue to limit its potential.

### Producers

Groundnut remains the backbone of smallholder farming in Senegal, with more than half of farmers cultivating it as their main cash crop. For many, it is more than just a commodity for oil mills, it provides food, seed, cash, and livestock feed. Yet producers face recurring obstacles. Access to quality seeds, fertilizers, and equipment is limited, droughts and soil depletion threaten yields, and weak farmer organizations reduce their bargaining power.

In the groundnut basin, farmers practice cereal–groundnut rotations in an extensive farming system. Over time, groundnuts have become the dominant crop in this landscape. Institutions such as ISRA and CERAAS promote improved seed varieties, but adoption is slow because of an underdeveloped seed system and insufficient policy support. Some digital platforms, like SEN LOUMA and SAIDA, now help farmers check market prices, but poor connectivity and lack of smartphones make them inaccessible to many. In places like Thiel, farmers often receive little or no technical support at all.

Each harvest, farmers set aside a portion of their crop for seeds, keep some for-household consumption, and sell the rest. Groundnut by-products such as husks and cakes are important source for feeding animals used for plowing, transport, and fattening, linking crop production with livestock keeping. In the absence of proper storage facilities and due to urgent cash needs, farmers are forced to sell grains immediately after harvest, when prices are lowest, sometimes as little as 250 CFA francs per kilogram. This cycle leaves producers vulnerable, reinforcing their dependence on groundnut even as it exposes them to significant risks.

### Input suppliers

The supply of inputs for groundnut production in Senegal is shaped largely by state subsidies. The government provides subsidized seeds, fertilizer, and farm equipment, and extends credit for input purchases. A major share of national agricultural subsidies is absorbed by the groundnut sector, but this heavy bias distorts incentives for other crops like millet and sorghum.

**Seed supply** is one of the weakest links. Although seeds are subsidized, they are often insufficient in volume and poor in quality. Seed supply comes from ISRA, private traders, “bana-bana,” and grassroots organizations. However, insufficient availability and distribution inefficiencies limits its reach. Cooperatives lack resources to buy quality seed on time, while private operators tend to market low-quality “off-the-shelf” seed that is more profitable than certified seed. The state’s seed control division (DISEM) lacks staff and infrastructure to enforce quality checks, which enables abuse of subsidies. Some large producers, often political or religious elites, obtain higher quotas of subsidized seeds without genuine farming activities. Such poor targeting results in smallholders poor access to improved varieties.

**Equipment supply** is equally skewed. Farmers rely mainly on renting tractors at negotiated rates. While many producers request government support, subsidized tractors often go to large producers, politicians, or marabouts. Small-scale farmers mostly access hand seeders, subsidized at 60%, yet even these remain too expensive for many and not available to all farmers. The shortage of seeders is critical given the short sowing window; delays in planting directly reduce yields. Even when organized in associations, smallholders struggle to access equipment on time.

**Fertilizer supply** is dominated by state distribution. Subsidized fertilizer, often just 4–5 kg per producer, is allocated through village chiefs or other prominent leaders, who tend to prioritize large farmers or influential figures. Quantities are far below requirements, leading to poor productivity. Farmers frequently complain that state assistance is insufficient, inputs are of low quality, and distribution channels are opaque. Where available, fertilizer is often paid for in cash, though some producers settle in kind with institutions like ISRA, PAFA, or SONACOS.

**Financial services** are provided mainly by Banque Agricole (BA), formerly CNCAS, supported by credit unions such as PAMECAS, Crédit Mutuel, and IMCEC. Financing is used primarily for acquiring seeds, fertilizer, and other inputs. The state often intervenes with credit guarantees to facilitate seasonal loans. Oil processors and traders secure their financing from commercial banks like BICIS. Producers may access credit individually or collectively, but only if they belong to formal groups such as Economic Interest Groups (EIGs).

Overall, the input supply system for groundnut is heavily state led, but marked by inefficiency, misallocation, and inequity. Subsidies are substantial, yet the benefits fail to reach most small-scale producers who depend on groundnut for their livelihoods.

### Trader

Groundnut trade in Senegal is concentrated immediately after harvest, when most farmers sell their entire crop. Sales move through three main channels: private traders, SONACOS/SUNEOR SA a national oilseeds marketing company in Senegal, focused on processing peanuts and producing edible oils, and Chinese buyers. Prices are lowest at harvest, often between 200 and 250 CFA francs/kg, but rise gradually from March onward.

In the past, government-set prices through CINA guided the market. Today, the official price negotiated for SONACOS purchases has limited influence, as Chinese traders offer higher prices and attract much of the supply. This shift has weakened SONACOS’ role and created tensions. Farmers complain of low and unstable prices, while SONACOS and private industry express concern over policies that allow foreign buyers to dominate the trade.

The market remains focused on oilseed and meal, but over the past three decades there has been gradual diversification toward edible groundnut products. Despite this, trade remains highly volatile, shaped by international demand, foreign competition, and the limited bargaining power of small farmers who are forced to sell quickly after harvest.

### Processor

Groundnut processing in Senegal is carried out by a mix of semi-industrial units and informal, family-based artisanal processors. Together they form an important link in the value chain, providing products for both household use and local markets. Raw materials are usually purchased from weekly markets (louma), and processed products are sold in surrounding villages and towns.

Artisanal processors produce items such as *noflaye* (groundnut powder for cooking), groundnut leg, and small amounts of oil. For *noflaye*, smaller groundnuts are preferred because larger kernels yield more oil than powder. Processing is mostly manual, with little access to machinery. For activities such as boiling groundnuts, processors often hire machine owners, but poor handling, overcooking or undercooking, reduces product quality. Despite these constraints, the products are generally considered good, since processors can select higher-quality kernels from local markets.

On the industrial side, SONACOS has historically dominated oil extraction. However, inefficiencies in its operations, combined with strong competition from China and India, have led to a decline in crude oil production. While Senegal continues to focus heavily on oilseed crushing, global competitors are diversifying into edible snacks and protein supplement products, creating new markets where Senegal remains underrepresented.

Processors face recurring challenges: irregular supply due to fluctuating exports, poor-quality raw material affecting outputs, lack of automation, and limited access to credit or technical support. Yet the sector holds significant potential. Promoting small oil mills, improving access to modern equipments, and encouraging diversification beyond oil into snacks and protein supplements could transform groundnut processing into a driver of rural industrial growth.

### Exporters

In 2023, Senegal’s groundnut export sector demonstrated resilience amid evolving global dynamics. The country exported approximately 247,580 metric tons of groundnuts between January and April 2025, with China absorbing more than 80% of these exports. This surge in exports is attributed to the lifting of the export tax on unprocessed groundnuts and the increased presence of Chinese importers in the market.

Despite this growth, Senegal’s share in global groundnut oil exports remains modest. In 2023, the country exported crude groundnut oil valued at $6.3 million, with key destinations including the United Kingdom, Italy, and Switzerland. However, this represents a small fraction of the global market, where countries like China and India dominate production and export volumes.

Senegal continues to maintain a strong position in the export of groundnut meal, thanks to SONACOS’ aflatoxin treatment processes. In 2013/14, the country held a 20% share of the global groundnut meal market, second only to Argentina.

However, the sector faces challenges, including competition from other African producers like Nigeria, Sudan, Ghana, and Mali, and policy shifts such as the temporary suspension of seed exports in 2015 and the introduction of an export tax in 2016, which have impacted producer prices.

Appendix Figure 1: Groundnut Value Chain in Senegal

#### The Domestic Market

Senegal’s domestic groundnut market has evolved alongside its export sector, primarily serving local consumption. Historically, households consumed locally refined groundnut oil. Vegetable oils entered the market in 1980/81, and today, industrially refined groundnut oil, mainly produced by CAIT, represents only about 5% of the domestic refined oil market (150–160,000 liters per year, excluding artisanal *seggal* oil). *Seggal* oil, though widely consumed, remains poorly understood; the government recognizes its economic significance as well as public health risks, particularly potential aflatoxin contamination due to inadequate detoxification.

#### Market Mechanisms and Price Determination

Producers typically organize sales through local collection points, sometimes transporting products themselves, which increases their marketing costs. Official collection and price systems are managed by CNIA, coordinating sales points and setting producer prices. However, an informal market, centered in Touba, dominates domestic trade. Producers prefer this system for its immediate cash payments, access to all groundnut by-products (fresh/dry pods, shelled seeds, and sorting residues), and the mobility of street vendors (*bana-bana*). Private operators exploit this system, purchasing groundnuts early or at weekly markets below official prices, capturing large margins due to both official rules and opportunistic practices (the “Mbappat” phenomenon).

Sales may also occur directly from fields or through producers’ associations, who contract with buyers such as COPEOL, SENAGRO (Chinese), or SONACOS (SUNEOR). Historically, price setting involved pre-harvest negotiations under colonial regimes, limiting producers’ options. Between 2006 and 2018, official producer prices averaged 25% above international reference prices (1). The government maintains a dual price system: the official producer price and the factory price set by processors. Opportunistic private operators sometimes exploit the *bana-bana* network to purchase at lower-than-official prices. Quality losses occur due to mandatory inspections and grading abatements, reducing producer returns.

Since 2016, the official producer price has been fixed at 210 CFA francs/kg, including the 2019/2020 marketing year. Local sales to oil companies are estimated at 346,853 tons annually, involving actors such as SONACOS SA, COPEOL, WAO, CAIT, and CNIA (55). This price policy protects producers from fluctuations in global markets, as seen during 2014 and 2018 when world demand for groundnut oil declined.

Privatization of SONACOS, Senegal’s largest buyer of groundnut shells and producer of crude oil, led to pressure on purchase prices, pushing producers toward more attractive buyers. Artisanal processors and Chinese companies now dominate these alternative markets, purchasing local production for export to China for processing. Groundnut product exports increased after China entered the market in 2014, reaching 147,000 tons in 2018, mostly as grains and only 25% as oil (1).

The domestic market thus functions as a complex mix of formal and informal channels, balancing producer protection, artisanal processing, and growing international trade, while remaining constrained by quality issues, price volatility, and structural inefficiencies.

## References

[1] FAO. *Suivi Des Politiques Agricoles et Alimentaires Au Sénégal 2021.* FAO (2021).

[2] FIDA. *L’avenir de l’agriculture au Sénégal: 2030-2063. Hub FIDA Afrique de l’Ouest.* FIDA (2020). p. 1–33.

[3] Georges N Fang S Beckline M Wu Y. Potentials of the groundnut sector towards achieving food security in Senegal. *Open Access Library J.* (2016) 3:1–13. 10.4236/oalib.1102991

[4] Gautreau J De Pins O. Groundnut production and research in Senegal. *Int Workshop Groundnuts.* Patancheru (AP): International Crops Research Institute for the Semi-Arid Tropics (1980). 274 p.

[5] Akobundu E. *Farm-Household Analysis of Policies Affecting Groundnut Production in Senegal.* Doctoral dissertation, Virginia Tech (1997).

[6] Toure K Diatta P Stone A Mbaye T Kostandini G Mills BF. *Groundnut Production Constraints and Opportunities for Young Adults in the Senegalese Groundnut Basin.* Athens (GA): Feed the Future Peanut Innovation Lab (2021).

[7] Reardon T Hesey P Timmer CP Berdegué J. Inclusive value chain transformation for rural development: conceptual framework and evidence. *Ann Rev Res Econ.* (2021) 13:335–58.

[8] Béné C Oosterveer P Lamotte L Brouwer ID de Haan S Prager SD et al. When food systems meet sustainability: current narratives and implications for actions. *World Dev.* (2019) 113:116–30. 10.1016/j.worlddev.2018.08.011

[9] Béné C Wood RG Newsham A Davies M. Resilience: new utopia or new tyranny? Reflection about the potentials and limits of the concept of resilience in relation to vulnerability reduction programmes. *IDS Work Pap.* (2012) 2012:1–61. 10.1111/j.2040-0209.2012.00405.x

[10] Folke C Carpenter SR Walker B Scheffer M Chapin T Rockström J. Resilience thinking: integrating resilience, adaptability and transformability. *Ecol Soc.* (2010) 15:20. 10.5751/ES-03610-150420

[11] FAO. *Groundnut Market Review: Trends and Outlook.* Food and Agriculture Organization of the United Nations (2023).

[12] Cutter SL Barnes L Berry M Burton C Evans E Tate E et al. A place-based model for understanding community resilience to natural disasters. *Glob Environ Change.* (2008) 18:598–606. 10.1016/j.gloenvcha.2008.07.013

[13] Meuwissen MPM Feindt PH Spiegel A Termeer CJAM Mathijs E de Mey Y et al. A framework to assess the resilience of farming systems. *Agric Syst.* (2019) 176:102656. 10.1016/j.agsy.2019.102656

[14] Wigboldus S Klerkx L Leeuwis C Schut M Muilerman S Jochemsen H. Systemic perspectives on scaling agricultural innovations: a review. *Agron Sustain Dev.* (2016) 36:46. 10.1007/s13593-016-0380-z

[15] Glover D Sumberg J Ton G Andersson J Badstue L. Rethinking technological change in smallholder agriculture. *Outlook Agric.* (2019) 48:169–80. 10.1177/0030727019864978

[16] Kaplinsky R Morris M. *A Handbook for Value Chain Research.* International Development Research Centre (2001). Available online at: https://www.ids.ac.uk/ids/global/pdfs/VchNov01.pdf (Accessed May 12, 2026)

[17] Gereffi G Humphrey J Sturgeon T. The governance of global value chains. *Rev Int Polit Econ.* (2005) 12:78–104. 10.1080/09692290500049805

[18] Ponte S Noe C Pallotti Corona L. Trading and value chain governance in the global cocoa–chocolate sector. *Geoforum.* (2019) 106:131–42. 10.1016/j.geoforum.2019.08.005

[19] Humphrey J Schmitz H. How does insertion in global value chains affect upgrading in industrial clusters? *Reg Stud.* (2002) 36:1017–27. 10.1080/0034340022000022198

[20] Gibbon P Bair J Ponte S. Governing global value chains: an introduction. *Econ Soc.* (2008) 37:315–38. 10.1080/03085140802172656

[21] Lazzarini SG Chaddad FR Cook ML. Integrating supply chain and network analyses: the study of netchains. *J Chain Netw Sci.* (2001) 1:7–22. 10.3920/JCNS2001.x002

[22] Ton G Bijman J Oorthuizen J editors. *Producer Organisations and Market Chains: Facilitating Trajectories of Change in Developing Countries.* Leiden (NL): Brill (2023).

[23] Sterman JD. *Business Dynamics: Systems Thinking and Modeling for a Complex World*. Boston, MA: Irwin/McGraw-Hill (2000). 982 p.

[24] Meadows DH. *Thinking in Systems: A Primer.* White River Junction, VT: Chelsea Green Publishing (2008). Available online at: https://research.fit.edu/media/site-specific/researchfitedu/coast-climate-adaptation-library/climate-communications/psychology-amp-behavior/Meadows-2008.-Thinking-in-Systems.pdf (Accessed May 12, 2026).

[25] Bolwig S Ponte S du Toit A Riisgaard L Halberg N. Integrating poverty and environmental concerns into value chain analysis: a strategic framework and practical guide. *Dev Policy Rev.* (2022) 40:e12605. 10.1111/dpr.12605

[26] Rich KM Ross RB Baker AD Negassa A. Quantifying value chain analysis in the context of livestock systems in developing countries. *Food Policy.* (2011) 36:214–22. 10.1016/j.foodpol.2010.11.018

[27] FAO. *The State of Food and Agriculture 2023: Revealing the True Cost of Food to Transform Agrifood Systems.* Rome: Food and Agriculture Organization of the United Nations (2023).

[28] Grabs J Ponte S. The evolution of power in the global coffee value chain and production network. *J Econ Geograp.* (2019) 19:803–28. 10.1093/jeg/lbz007

[29] Springfield Centre. *The Operational Guide for the Making Markets Work for the Poor (M4P) Approach.* 2nd ed. Durham: Springfield Centre (2014). Available online at: https://www.springfieldcentre.com/wp-content/uploads/2014/09/2014-09-M4P-Operational-Guide-with-watermark1.pdf (Accessed May 12, 2026)

[30] Richardson GP. Reflections on the foundations of system dynamics. *Syst Dyn Rev.* (2011) 27:219–43. 10.1002/sdr.462

[31] Moores D Hunter A. *Inclusive Market Systems Development: Sustainable Growth for Everyone.* Sydney: World Vision Australia (2018). Available online at: https://www.worldvision.com.au/docs/default-source/publications/aid-trade-and-mdgs/wva.inclusive-market-systems-development-paper.final.pdf (accessed May 12, 2026).

[32] Niles MT Häberli I Berthet E Fresco LO. Food systems research priorities for a sustainable future. *Nature Food.* (2021) 2:383–9.37118230

[33] Pruyt E Kwakkel JH. System dynamics in practice: from problem framing to policy design in complex systems. *Syst Res Behav Sci.* (2022) 39:651–68. 10.1002/sres.2873

[34] Simmonds H Rouwette E Smits M. Participatory modelling for agri-food system transformation: lessons from group model building in West Africa. *Agric Syst.* (2023) 206:103600.

[35] Cissé D. *Chinese Involvement in the Senegalese Peanut Trade: Threat to Local Markets and Processing Industries?.* Stellenbosch (South Africa): Centre for Chinese Studies, Stellenbosch University (2014).

[36] Rouwette E Bleijenbergh I Vennix J. Group model building for policy analysis: a review of applications and developments. *Policy Sci.* (2022) 55:229–52.

[37] Fresia A Rouwette E Vennix J. Boundary management in group model building: how facilitators manage the participation process. *Syst Dyn Rev.* (2023) 39:4–29. 10.1002/sdr.1710

[38] Vennix JA. Group model-building: tackling messy problems. *Syst Dyn Rev.* (1999) 15:379–401. 10.1002/(SICI)1099-1727(199924)15:4<379::AID-SDR179>3.0.CO;2-E

[39] Hovmand PS. Group model building workshop and facilitation. In: Hovmand PS editor. *Community Based System Dynamics.* New York (NY): Springer (2014). p. 61–76. 10.1007/978-1-4614-8763-0_6

[40] Black LJ Andersen DF. Using visual representations as boundary objects to resolve conflict in collaborative model-building approaches. *Syst Res Behav Sci.* (2012) 29:194–208. 10.1002/sres.2106

[41] Black LJ. When visuals are boundary objects in system dynamics work. *Syst Dyn Rev.* (2013) 29:70–86. 10.1002/sdr.1496

[42] Rose J Homa L Hovmand P Kraus A Burgess K Biswas A et al. Boundary objects for participatory group model building of agent-based models. *2015 48th Hawaii Int Conf Syst Sci.* Piscataway (NJ): IEEE (2015). p. 2950–9.

[43] Hovmand PS Andersen DF Rouwette E Richardson GP Rux K Calhoun A. Group model-building ‘scripts’ as a collaborative planning tool. *Syst Res Behav Sci.* (2012) 29:179–93. 10.1002/sres.2105

[44] Andersen DF Richardson GP Vennix JAM. Group model building: adding more science to the craft. *Syst Dyn Rev.* (1997) 13:187–201. 10.1002/(SICI)1099-1727(199722)13:2<187::AID-SDR124>3.0.CO;2-O

[45] Sterman J. *Business Dynamics: Systems Thinking and Modeling for a Complex World.* Boston, MA: McGraw-Hill (2002).

[46] Yessoufou AND Kumar S Houessionon P Worou ON Wane A Whitbread A. Vulnerability and resilience in the face of climate changes in Senegal’s drylands: measurement at the household level and determinant assessment. *Front Clim.* (2024) 6:1330025. 10.3389/fclim.2024.1330025

[47] Grovermann C Wossen T Margraf J Oyakhilomen O Abdoulaye T. Seed system interventions and food security among smallholder farmers in sub-Saharan Africa. *Food Policy.* (2021) 103:102121. 10.1016/j.foodpol.2021.102121

[48] Traore B Descheemaeker K van Wijk M Corbeels M Supit I Giller KE et al. Modelling climate change effects on groundnut in West Africa: yield trends and adaptation options. *Field Crops Res.* (2021) 263:108059. 10.1016/j.fcr.2021.108059

[49] Zougmoré R Partey S Ouédraogo M Torquebiau E Campbell B. Facing climate variability in sub-Saharan Africa: analysis of climate-smart agriculture opportunities to manage climate-related risks. *Cahiers Agri.* (2021) 30:17. 10.1051/cagri/2021001

[50] Meinzen-Dick R Quisumbing A Doss C Theis S Women’s land rights as a pathway to poverty reduction: framework and review of available evidence. *Agric Syst.* (2019) 172:72–82. 10.1016/j.agsy.2018.01.014

[51] Galiè A Teufel N Korir L Baltenweck I Webb Girard A Dominguez-Salas P et al. The women’s empowerment in livestock index. *Soc Indic Res.* (2019) 142:799–825. 10.1007/s11205-018-1934-z

[52] Ndour NYB Diallo MD Guisse A. Post-harvest losses and aflatoxin contamination in groundnut value chains in Senegal: constraints and opportunities. *J Stored Prod Res.* (2022) 97:101953. 10.1016/j.jspr.2022.101953

[53] Sultan B Defrance D Iizumi T. Evidence of crop production losses in West Africa due to historical global warming in two crop models. *Sci Rep.* (2020) 10:1772. 31492929 10.1038/s41598-019-49167-0 PMC6731230

[54] Minot N Daniels L. Impact of global commodity price volatility on rural incomes and food security in Africa. *World Dev.* (2022) 150:105734. 10.1016/j.worlddev.2021.105734

[55] Agence Nationale de la Statistique et de la Démographie (ANSD). *Situation Economique et Sociale du Sénégal en 2019*. Dakar: ANSD (2020).

